# A masked, placebo-controlled, randomized clinical trial evaluating safety and the effect on cardiac function of low-dose rapamycin in 17 healthy client-owned dogs

**DOI:** 10.3389/fvets.2023.1168711

**Published:** 2023-05-18

**Authors:** Brian G. Barnett, Sonya R. Wesselowski, Sonya G. Gordon, Ashley B. Saunders, Daniel E. L. Promislow, Stephen M. Schwartz, Lucy Chou, Jeremy B. Evans, Matt Kaeberlein, Kate E. Creevy

**Affiliations:** ^1^Department of Small Animal Clinical Sciences, School of Veterinary Medicine and Biomedical Sciences, Texas A&M University, College Station, TX, United States; ^2^Department of Biology, University of Washington, Seattle, WA, United States; ^3^Department of Laboratory Medicine and Pathology, University of Washington, Seattle, WA, United States; ^4^Epidemiology Program, Fred Hutchinson Cancer Center, Seattle, WA, United States

**Keywords:** diastolic function, aging, age-related decline, echocardiography, geroscience

## Abstract

**Introduction:**

Geroscience studies of low-dose rapamycin in laboratory species have identified numerous benefits, including reversing age-related cardiac dysfunction. Cardiovascular benefits have been observed in dogs with 10 weeks of treatment, raising questions about possible benefits and adverse effects of long-term use of low-dose rapamycin. The objectives of this study were to assess the impact of 6 months of low-dose rapamycin on echocardiographic indices of cardiac function in healthy dogs and to document the occurrence of adverse events.

**Methods:**

Seventeen client-owned dogs aged 6–10 years, weighing 18–36 kg, and without significant systemic disease were included in a prospective, randomized, placebo-controlled, masked clinical trial. Low-dose rapamycin (0.025 mg/kg) or placebo was administered three times per week for 6 months. Baseline, 6-month, and 12-month evaluation included physical examination, cardiology examination, and clinicopathology. Three-month evaluation included physical examination and clinicopathology. Owners completed online questionnaires every 2 weeks.

**Results:**

There were no statistically significant differences in echocardiographic parameters between rapamycin and placebo groups at 6 or 12 months. No clinically significant adverse events occurred. In 26.8% of the bi-weekly surveys owners whose dogs received rapamycin reported perceived positive changes in behavior or health, compared to 8.1% in the placebo group (*p* = 0.04).

**Discussion:**

While no clinically significant change in cardiac function was observed in dogs treated with low-dose rapamycin, the drug was well-tolerated with no significant adverse events.

## 1. Introduction

Normal cardiac aging results in multiple structural and functional changes over time as well as increased incidence of age-related cardiovascular diseases. In humans, dilation of the left atrium, increased left ventricular wall thickness, and diastolic dysfunction are well-recognized age-related changes (1–4). In dogs, echocardiographic evidence of diastolic dysfunction associated with age has also been demonstrated, including increased isovolumic relaxation time in dogs >10 years of age and decreased mitral inflow E to A wave ratios (E:A) in dogs >6 years of age when compared to dogs <2 years of age (5). On a cellular level, upregulation of inflammatory cytokines that promote cardiac fibrosis and hypertrophy, decreased macroautophagy and increased oxidative stress are all proposed mechanisms of cardiac aging (3, 6).

Rapamycin is a macrocylic lactone molecule first identified from soil samples obtained on Easter Island. It is naturally produced by the bacterium *Streptomyces hygroscopicus*, and was first studied for its potent antifungal activity. It was later found to also inhibit growth of mammalian cells and was developed clinically for use as an anti-cancer and immune suppressive agent. Rapamycin (sirolimus) was approved by the FDA in 1999 to prevent renal transplant rejection and, along with rapamycin derivatives (rapalogs), has been used in organ transplant medicine for more than two decades. Additionally, rapamycin or rapalogs have been FDA approved for use in cardiac stents to prevent restenosis, for use in tuberous sclerosis complex disorders, and for use in some forms of cancer (7, 8).

Rapamycin is a mechanistic target of rapamycin complex I (mTORC1) inhibitor. mTOR signaling plays a major role in regulation of cell growth, nutrient response, proteostasis, and survival (9–11). Recent work suggests that rapamycin impacts the aging process at a cellular level by inducing macroautophagy, altering mRNA translation, modifying nutrient sensing, decreasing sterile inflammation, and improving stem cell function (11–13). Researchers have increased lifespan in yeast (14), *Caenorhabditis elegans* (15), and *Drosophila melanogaster* (16) through modification of mTOR signaling by various means. Rodent rapamycin studies have shown numerous benefits, including increased lifespan (17–23), improved cognitive and muscle function (22, 24, 25), reduced tumorigenesis (26), prevention of retinopathy (27), restoration of stem cell function (9, 28), reversal of immune declines (9), reversal of periodontal disease (29, 30), improved kidney function (31), preservation of tendon (32), improved intestinal function and reduced gut dysbiosis (22, 28), preservation of ovarian function (33) protection against hearing loss (34), and reversal of age-related cardiac dysfunction, specifically by improving indices of systolic function [ejection fraction (EF), fractional shortening (FS), and left ventricular end systolic dimensions] and decreasing age related cardiac hypertrophy (6, 35, 36). Rapamycin was recently accessed in a clinical trial as an adjuvant therapy for appendicular osteosarcoma, but no significant improvement in median disease-free interval or overall survival were observed (37).

At dosages intended to mitigate age-related changes, adverse events associated with rapamycin are uncommon in rodents, and include hyperlipidemia, gonadal atrophy, and cataract formation, as well as altered glucose homeostasis with chronic use (19, 38). In studies of low-dose rapamycin and its derivatives in elderly humans, adverse events are generally mild and transient, and include mouth ulcers, diarrhea, elevated cholesterol, and headache (39, 40). The few *in vivo* studies in dogs evaluating rapamycin at non-immunosuppressive doses have shown it is well-tolerated with low incidence of adverse events (41, 42).

Additional work is needed to fully elucidate the cellular mechanisms by which rapamycin exerts these reported effects. Previously, our group investigated low-dose rapamycin and placebo treatments in healthy, middle-aged dogs over a 10-week period, with the primary goal of establishing the safety of low-dose rapamycin in healthy pet dogs. Cardiac endpoints were also assessed because of the robust evidence of rapamycin’s mitigation of age-related cardiac functional decline in mice (6, 36, 43). The study period was only 10 weeks, and all dogs had normal echocardiograms at enrollment. Interestingly, this trial found that FS improved in treated dogs, although a statistically significant change in EF was not detected, over the 10-week study period. Additionally, the E/A ratio was statistically significantly higher in treated dogs compared to placebo, and no clinically meaningful adverse events were observed over the 10-week treatment period (44). The results of this small and short-term trial prompted the decision to undertake a follow-up study over a longer period of time.

The advantages of further evaluating the potential benefits of rapamycin and other geroscience therapeutics in companion dogs are numerous. These studies could validate therapies that reduce or delay age-related disease and increase healthspan in companion dogs. Furthermore, conclusions from geroscience investigations in dogs have high translational potential to human geroscience, as companion dog aging draws close parallels to human aging; humans and companion dogs share common environments, receive comparable medical care, and experience many similar age-related diseases (43, 45, 46).

The major objectives of this study were to assess the effect of 6 months of low-dose rapamycin treatment on baseline indices of diastolic and systolic cardiac function and to document occurrence of adverse events in healthy, middle-aged companion dogs. We hypothesized that rapamycin would improve echocardiographic indices of diastolic function in treated dogs compared to placebo-treated controls over a 6-month period. We also hypothesized that no treated dogs would demonstrate adverse events requiring medical intervention. Assessment of the impact of low-dose rapamycin on routine clinical pathology was a secondary objective.

## 2. Materials and methods

### 2.1. Trial design

This study was a prospective, randomized, placebo-controlled double-masked trial conducted at the Texas A&M University (TAMU) Veterinary Medical Teaching Hospital (VMTH).

### 2.2. Dogs

#### 2.2.1. Inclusion criteria

To be eligible, dogs were required to be between the ages of 6 and 10 years and have a body weight > 18 and < 36 kg. A negative heartworm antigen test was required, as was documentation of consistent heartworm prevention administration for the preceding 6 months and the owner’s willingness to continue heartworm prevention. Documentation of appropriate vaccination status in concordance with the 2017 AAHA Canine Vaccination Guidelines (47) was also required, as well as the owner’s willingness to continue vaccination. On physical examination, a grade II/VI or softer systolic murmur was permitted.

#### 2.2.2. Exclusion criteria

Dogs were excluded from the study if any of the following systemic diseases were present in their history or identified at the screening/baseline examination: diabetes mellitus, hyperadrenocorticism, hypothyroidism, renal disease, liver disease, chronic gastrointestinal disease, respiratory disease, neurological disease, systemic hypertension, cardiac arrhythmia other than sinus arrhythmia, and cardiac disease other than ACVIM Stage B1 myxomatous mitral valve degeneration (48). A diagnosis of occult dilated cardiomyopathy was made if the normalized left ventricular internal diameter at end-systole was >1.26 (49) in combination with a FS <20% and/or an EF <40% (50). Dogs were also excluded if they exhibited poor tolerance of examination and venipuncture, had a SBP greater than 160 mmHg, had a poor echocardiographic scanning window, or if another dog in the same household was enrolled.

#### 2.2.3. Enrollment process

Owners nominated their dogs through an online eligibility questionnaire that requested basic demographic, health history, and temperament information and that also provided background on the study design and commitments required. Owners who indicated understanding of the trial and availability for four VMTH visits, and whose dogs were the correct age (6–10 years), correct weight (18–36 kg), and described as being in good health and cooperative for veterinary visits and medication administration, were invited to submit their dogs’ comprehensive veterinary medical records. Records in any format were accepted, and a minimum of 3 years of records was required. Submitted records were evaluated for the presence of exclusion criteria.

Owners of dogs with no exclusionary findings in their medical records were invited to schedule a screening examination. For those dogs who were enrolled, this also served as the baseline examination. At the time of the appointment, the study was described in detail to the owners, and they were allowed to ask questions before reviewing and signing an Informed Owner Consent form. All procedures and forms were reviewed and approved by the TAMU College of Veterinary Medicine and Biomedical Sciences’ Institutional Animal Care and Use Committee and Clinical Research Review Committee under protocol number 2017–0125.

The screening evaluation included a physical examination, complete blood count, serum chemistry profile, urinalysis, total thyroxine level (TT4) plus free thyroxine and thyroid stimulating hormone if TT4 was low, heartworm antigen test, systolic arterial blood pressure (SBP) measurement, electrocardiogram (ECG), and echocardiogram. If no exclusion criteria were identified after this process, dogs were considered eligible for the study.

### 2.3. Randomization and masking

Dogs who fulfilled all enrollment criteria were enrolled and randomized using a schedule designed and administered by the TAMU VMTH pharmacist. A block randomization schedule was used with blocks of four and a 1:1 allocation ratio to either the rapamycin group or placebo group.

### 2.4. Trial medication

Rapamycin [Dr. Reddy’s (1 mg), Princeton, NJ; ZyGenerics (0.5 mg), Pennington, NJ] was formulated into capsules containing whole or half tablets to create capsules containing 0.5, 0.75, or 1.0 mg of rapamycin. Placebo (lactose; PCCA, Houston, TX) capsules were created to be identical in appearance. Dogs in the treatment group were given 0.025 mg/kg rapamycin per dose, rounded to the nearest 0.25 mg capsule. Owners were instructed to give the study medication on Monday, Wednesday, and Friday mornings. Owners were provided with study medication logs to document administration. Administration of rapamycin and placebo was continued for a period of 6 months. Owners returned pill vials to the TAMU VMTH pharmacy at each visit, and leftover capsules were counted.

### 2.5. Clinical evaluation

Following the screening/baseline examination, dogs were examined at 3 and 6 months during the treatment period. A final examination was performed at 12 months (6 months after discontinuation of study medication) to determine whether any changes induced by rapamycin therapy persisted beyond the treatment period. The 3-month examination included history, physical examination, complete blood count, serum chemistry profile, urinalysis, and SBP measurement, without cardiology examination. The 6- and 12-month examinations were identical and included history, physical examination, complete blood count, serum chemistry profile, urinalysis, SBP measurement, ECG, and echocardiogram.

### 2.6. Systolic arterial blood pressure

All SBP measurements were taken by a trained clinician or technician prior to physical examination and other diagnostics. Dogs were allowed 10–15 min of acclimatization in a quiet environment before measurements were taken. The SBP measurements were taken on the antebrachium with a Doppler unit, utilizing a cuff with a diameter approximately 40% of the extremity circumference. Body position, cuff size, and cuff location were recorded. The first measurement was discarded, and three consecutive values were recorded and averaged. If consecutive readings varied by >20%, the cuff was repositioned, and the measurements repeated until ≤20% variation was observed.

### 2.7. Electrocardiography

Standard six-lead ECGs were performed with dogs positioned in right lateral recumbency. Three-minute ECG traces were recorded. All dogs were conscious and unsedated for the recording. One of three board-certified veterinary cardiologists (SW, SG, AS) evaluated the ECG traces for the presence of arrhythmias. Because drug-induced QT prolongation is a potential adverse event associated with diverse medications (51), three corrected QT intervals (QTc) were also calculated and averaged (52).

### 2.8. Echocardiography

Standard echocardiography including two-dimensional, M-mode and Doppler imaging was performed by a board-certified cardiologist using a Vivid E95 Vet 2.0 (GE Healthcare, Chicago, IL, United States) with dogs unsedated and gently restrained in right and left lateral recumbency. Each measurement was taken in triplicate and the averages of these measurements were recorded. The following measurements were obtained: left atrial to aortic root ratio (LA:Ao) (53), left atrial major axis diameter obtained at end ventricular systole from a right parasternal long axis view (54), left ventricular internal dimension at end-diastole and end-systole obtained from short axis M-mode images at the level of the papillary muscles which were then normalized to body weight as LVIDdN and LVIDsN (49), normalized left ventricular free wall thickness at end diastole (LVPWdN) (49), left ventricular FS, left ventricular EF from a left apical four-chamber view using Simpson’s method of discs, peak E wave velocity, mitral inflow E to A wave ratio, isovolumic relaxation time (IVRT), peak aortic and pulmonic velocities (AV Vmax, PV Vmax) and aortic velocity time integral (AV VTI), as well as color Doppler investigation of all valves with presence and severity of any valvular insufficiencies noted. Each dog was grouped into a diastolic functional class for baseline, 6 and 12 month time points which were defined as: normal pattern (Class 1), relaxation delay pattern (Class 2), pseudonormal pattern (Class 3), and restrictive pattern (Class 4) (55).

### 2.9. Clinicopathologic evaluation

Peripheral blood, urine and fecal samples were collected from all dogs at each visit. Urine was collected by cystocentesis or catheterization if free catch was not successful. The TAMU VMTH Clinical Pathology Laboratory performed complete blood counts (Advia 120, Siemens Healthineers, Malvern, PA), serum chemistry profiles (Vitros 4,600, Ortho Clinical Diagnostics, Raritan, New Jersey), and urinalyses (Multistix 10SG urine strips, read on a Clinitek strip reader, both by Siemens Healthineers, Malvern, PA). At the baseline examination only, TT4 was measured by the TAMU Gastrointestinal Laboratory, and if that value was below the reference interval (<1.7 μg/dL), free thyroxine and thyroid stimulating hormone assays were performed (Immulite 2000 Xpi, Siemens Healthineers, Malvern, PA). The TAMU Clinical Immunology Service performed heartworm antigen testing (DiroCHEK Canine Heartworm Antigen Test Kit, Zoetis, Parsippany-Troy Hills, NJ) according to manufacturer instructions. Because all enrolled dogs resided in Texas, samples were submitted to the Texas A&M Veterinary Medical Diagnostic Laboratory to screen for exposure to the agent that causes Chagas disease (*Trypanosoma cruzi*) with immunofluorescent antibody tests. For this assay, serum samples were diluted 1:20 in phosphate buffered saline (PBS) and incubated on substrate slides (Trinity Biotech, Jamestown, NY) containing fixed *Trypanosoma cruzi* epimastigotes. The slides were then washed with PBS, and an anti-dog IgG secondary antibody conjugated with FITC (SeraCare/KPL, Milford, MA) was added to all sample wells. Following incubation with the secondary antibody, the slides were washed and examined under a fluorescent microscope. Sample wells exhibiting bright fluorescence of the fixed epimastigotes were considered positive for *T. cruzi* antibodies at the 1:20 dilution. Any samples testing positive at the 1:20 dilution were serially diluted twofold and tested again; the reciprocal of the highest dilution exhibiting a positive reaction was reported as the antibody titer. Blood and fecal samples were banked from each dog at each examination. Banked baseline and 6-month samples were analyzed by the Michigan State University Veterinary Diagnostic Laboratory to determine fasting insulin (Insulin RIA, Millipore Sigma, Burlington, MA), and glucose (AU680, Beckman Coulter, Brea, CA) concentrations and results were used in the homeostatic model assessment for insulin resistance (HOMA-IR) to estimate the current level of insulin resistance, as well as beta cell function (56, 57). At the conclusion of the study, banked baseline and 6-month samples were analyzed by the TAMU Gastrointestinal Laboratory to determine cardiac troponin (cTnI) concentrations using a three-site sandwich direct chemiluminometric immunoassay (TnI-Ultra platform Advia Centaur CP, Siemens Healthineers, Malvern, PA) (58).

### 2.10. At-home observation

Each owner was sent an automated online questionnaire every 2 weeks for the duration of the study. This questionnaire included forced-choice items addressing specific adverse events over the prior two-week period, forced-choice items addressing aspects of canine cognitive dysfunction, and free-text items addressing general negative or positive changes in behavior.

### 2.11. Statistical analysis

Baseline characteristics of the dogs were summarized by assigned treatment group (rapamycin vs. placebo). The primary intent-to-treat analysis method was calculation of the absolute difference in echocardiographic outcome measures between the treatment groups at each follow-up time point (6 and 12 months). The null hypothesis of no difference for each outcome measure was tested using two-sample *t*-tests. Due to the small sample size, only univariable comparisons between treatment groups were performed. The two-sided type 1 hypothesis testing threshold was set at 0.05 for assessments of treatment effect on each parameter. For echocardiographic parameters, the *p-*value was adjusted using the Holm-Bonferroni step-down method to account for multiple comparisons (59).

HOMA-IR scores were compared between treatment groups at baseline and 6 months using two-sample *t*-tests.

Owner responses on questionnaires were inspected for reports of adverse events; too few adverse events occurred to permit formal analyses. Owners answered questions about positive changes in behavior an average of 23 ± 3.1 times (mean ± 1 SD). We calculated the average positivity score for each owner, converting yes/no responses to the question regarding observed positive changes in behavior to 1/0, respectively, and taking the numeric average across all answers. We then compared mean positivity scores of rapamycin-treated and control groups using a two-sample two-tailed t-test. Free-text explanations of any observed changes were inspected informally for common themes.

## 3. Results

**Figure 1:**
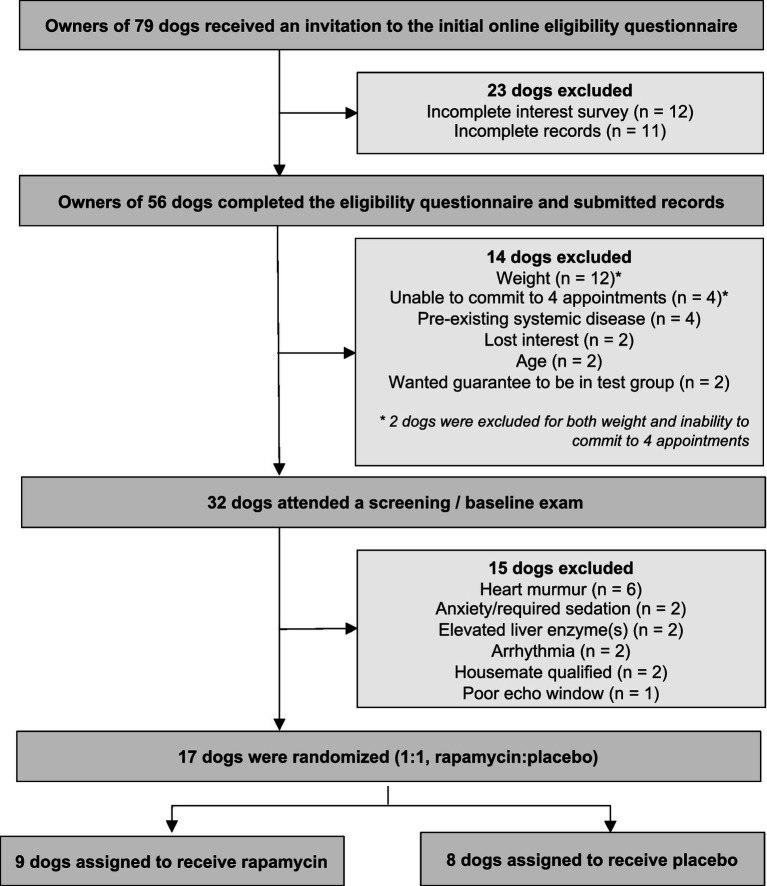
Flow diagram of enrollment and randomization process.

The owners of 79 dogs contacted the study investigators by email to inquire about the study and were sent an email reply including an invitation to the initial online eligibility questionnaire. Twenty-three owners either did not respond to the email or did not complete the initial online eligibility questionnaire. Fifty-six owners nominated their dogs by completing the initial online eligibility questionnaire. Of these, 39 dogs were excluded as previously reported (60) and 17 dogs were enrolled and randomly assigned to rapamycin or placebo groups. A flow diagram representing the enrollment and randomization process is depicted in Figure 1. There were no differences between groups in key characteristics at baseline (Table 1).

**Table 1:** Baseline characteristics of dogs in the two treatment groups in the trial population.

		Treatment groups	
Characteristic		Rapamycin (*n* = 9)	Placebo (*n* = 8)
Age at enrollment (years)		7.8 (1.0)	8.5 (1.7)
Sex and castration status	Male/castrated	4 (44.4)	2 (25.0)
	Female/spayed	4 (44.4)	6 (75.0)
	Female/intact	1 (11.1)	0 (0.0)
Breed category	Herding	2 (22.2)	4 (50.0)
	Sporting	4 (44.4)	2 (25.0)
	Terrier	1 (11.1)	0 (0.0)
	Working	2 (22.2)	0 (0.0)
	Other	0 (0.0)	2 (25.0)
Body condition score	4	3 (33.3)	0 (0.0)
	5	4 (44.4)	4 (50.0)
	6	2 (22.2)	4 (50.0)
Left atrial to aortic root ratio		1.16 (0.12)	1.19 (0.17)
Ejection fraction (%)		58.3 (11.3)	58.6 (8.1)
Fractional shortening (%)		30.1 (9.7)	32.2 (8.5)
E to A wave ratio		1.15 (0.30)	0.98 (0.27)

Continuous variables are reported as mean (standard deviation). Categorical variables are reported as number (%).

### 3.1. Clinical evaluation

Over the 12-month period of the study, the body weights of each dog varied by an average of 2.0 kg across the four visits. There was no association between treatment group and net gain or net loss. Over the 12-month period of the study, the SBP of each dog varied by an average of 31 mmHg across the four visits. Most dogs (12/17) showed an overall decline in SBP with subsequent visits, and no dog developed sustained hypertension (SBP ≥ 180 mmHg) during the study. One placebo-treated dog had SBP of 190 mmHg at the 3-month visit, which declined to 150 mmHg without treatment at the 6-month visit.

Abnormalities reported in the medical history by owners of dogs in the rapamycin group over the 12-month study period included decreasing willingness to exercise vigorously at agility competitions in one dog; apparent false pregnancy in one intact female dog; acute moist pyoderma in one dog; and diagnosis of a cutaneous mast cell tumor that was removed by the primary care veterinarian in one dog. Abnormalities reported in the medical history by owners of dogs in the placebo group over the 12-month study period included improvement in long-standing bilious vomiting syndrome in one dog; unilateral conjunctivitis that resolved with topical therapy in one dog; intermittent anal sacculitis in one dog; and declining vision in two dogs.

New abnormalities detected on physical examination over the 12-month study period included development of subcutaneous masses compatible with lipomas in eight dogs (*n* = 4 rapamycin); increasing stiffness on gait observation associated with pain and decreased range of motion on physical examination [hips (*n* = 2) or lumbosacral region (*n* = 1)] in a total of three dogs (*n* = 2 rapamycin); and a focal pruritic, crusted lesion on the nasal planum in one dog in the rapamycin group that persisted for 3 months and then resolved with no therapy.

One owner of a dog in the rapamycin group administered the study medication incorrectly (daily instead of three times per week) for a two-week period between the baseline visit and the 3-month recheck. There were no apparent adverse events from this dosing error. No other dosing errors were detected through inspection of owner medication logs or returned pill vials.

### 3.2. Electrocardiography

No new arrhythmias were diagnosed in any dogs during the 12-month study period. When analyzing the change in QTc from baseline to both the 6- and 12-month time points, there appeared to be a > 1% decrease associated with rapamycin treatment (Table 2). However, this result was not statistically significant at either time point (*p* = 0.304, and *p* = 0.800, respectively).

**Table 2:** Mean (standard deviation) echocardiographic values for rapamycin and placebo groups at baseline, 6  months, and 12 months into the study.

Outcome measure	Treatment group	Baseline	6 month mean (SD)	6 month effect*	*p*-value**	Adjusted *p*-value***	12 month mean (SD)	12 month effect*	*p*-value**	Adjusted *p*-value***
Left atrial to aortic root ratio	Rapamycin	1.159 (0.117)	1.132 (0.144)	−0.18 (−0.32, 0–0.04)	0.014	0.196	1.138 (0.112)	−0.166 (−0.321, −0.012)	0.037	0.481
	Placebo	1.195 (0.168)	1.315 (0.127)				1.304 (0.182)			
Left atrial major diameter^ (cm)	Rapamycin	3.58 (0.41)	3.69 (0.42)	0.06 (−0.33, 0.44)	0.757	1	3.74 (0.43)	0.292 (−0.113, 0.697)	0.145	1
	Placebo	3.42 (0.35)	3.63 (0.30)				3.45 (0.34)			
Ejection fraction (%)	Rapamycin	58.27 (11.86)	62.33 (13.96)	1.63 (−6.41, 9.67)	0.692	1	58.77 (7.29)	1.98 (−9.10, 12.99)	0.707	1
	Placebo	58.55 (8.10)	59.15 (10.00)				56.79 (13.47)			
Fractional shortening (%)	Rapamycin	30.08 (9.75)	30.98 (7.65)	−2.91 (−9.60, 3.78)	0.524	1	31.85 (6.12)	−0.513 (−7.062, 6.037)	0.87	1
	Placebo	32.21 (8.50)	33.89 (4.74)				32.37 (6.55)			
LVIDdN	Rapamycin	1.463 (0.093)	1.420 (0.093)	0.003 (−0.103, 0.110)	0.948	1	1.444 (0.131)	−0.026 (−0.180, 0.128)	0.721	1
	Placebo	1.456 (0.171)	1.417 (0.113)				1.470 (0.166)			
LVIDsN	Rapamycin	0.953 (0.146)	0.916 (0.133)	0.04 (−0.08, 0.16)	0.489	1	0.920 (0.145)	−0.014 (−0.055, 0.102)	0.856	1
	Placebo	0.921 (0.151)	0.876 (0.093)				0.934 (0.157)			
Peak E-wave velocity (m/s)	Rapamycin	0.65 (0.11)	0.70 (0.12)	−0.05 (−0.19, 0.01)	0.511	1	0.67 (0.10)	−0.004 (−0.113, 0.105)	0.938	1
	Placebo	0.66 (0.20)	0.74 (0.16)				0.68 (0.11)			
E to A wave ratio	Rapamycin	1.15 (0.30)	1.10 (0.22)	−0.05 (−0.29, 0.19)	0.688	1	1.19 (0.12)	0.201 (0.054, 0.347)	0.011	0.154
	Placebo	0.98 (0.27)	1.15 (0.24)				0.99 (0.16)			
IVRT (ms)	Rapamycin	83.08 (12.28)	79.39 (13.01)	−9.32 (−21.35, 2.72)	0.119	1	85.33 (13.42)	2.232 (−11.523, 15.988)	0.734	1
	Placebo	84.10 (9.74)	88.71 (9.77)				83.10 (13.12)			
AV Vmax (m/s)	Rapamycin	1.45 (0.26)	1.57 (0.25)	0.02 (−0.31, 0.28)	0.89	1	1.44 (0.20)	0.003 (−0.203, 0.196)	0.972	1
	Placebo	1.49 (0.24)	1.59 (0.32)				1.44 (0.19)			
AV VTI (cm)	Rapamycin	17.76 (4.66)	16.50 (1.87)	−1.14 (−7.45, 0.82)	0.018	0.234	17.85 (4.60)	0.743 (−3.200, 4.685)	0.694	1
	Placebo	17.63 (3.21)	20.64 (4.24)				17.10 (2.62)			
PV Vmax (m/s)	Rapamycin	0.89 (0.22)	1.02 (0.24)	0.08 (−0.19, 0.34)	0.544	1	0.95 (0.17)	0.068 (−0.091, 0.226)	0.375	1
	Placebo	0.83 (0.22)	0.94 (0.27)				0.88 (0.13)			
LVPWdN	Rapamycin	0.462 (0.073)	0.485 (0.044)	−0.010 (−0.044, 0.062)	0.7	1	0.452 (0.042)	−0.016 (−0.059, 0.026)	0.418	1
	Placebo	0.473 (0.061)	0.475 (0.057)				0.469 (0.039)			
QTc (ms)	Rapamycin	206.57 (6.48)	206.99 (13.82)	−7.19 (−21.06, 7.22)	0.304	1	196.13 (15.62)	−3.22 (−23.38, 29.82)	0.8	1
	Placebo	216.10 (17.32)	214.17 (14.01)				192.91 (33.67)			

AV Vmax, peak aortic velocity; AV VTI, aortic velocity time integral; IVRT, isovolumic relaxation time; LVIDdN, normalized left ventricular internal dimension in diastole; LVIDsN, normalized left ventricular internal dimension in systole; LVPWdN, normalized left ventricular free wall thickness at end diastole; PV Vmax, peak pulmonic velocity.

^Does not include baseline measurement from dog 17, which was lost due to software error.

*Per unit difference in the measure (95% confidence interval) associated with rapamycin (compared to placebo).

**2-sided test of the null hypothesis of no difference between the rapamycin group compared to placebo.

***2-sided test of the null hypothesis of no difference between the rapamycin group compared to placebo, adjusted for multiple comparisons using the Holm-Bonferroni step-down method.

### 3.3. Echocardiography

Table 2 displays the results for each echocardiographically-defined outcome measure recorded at the 6-month follow-up visit. While several outcomes appeared to show that treatment with rapamycin was associated with increases (EF) or decreases (FS, IVRT, AV VTI) in certain parameters by >1%, none of these results were statistically significant when accounting for the large number of outcomes being compared.

Table 2 also displays the results for each echocardiographically-defined outcome measure at 12 months. Again, while two outcomes appeared to show that treatment with rapamycin yielded increases (EF) or decreases (IVRT) in certain parameters by >1% neither of these were statistically significant after accounting for multiple comparisons. Mean values for each treatment group for all echocardiographic parameters collected are displayed in Table 2. The number of dogs in each diastolic functional class for each treatment and time point is displayed in Table 3.

**Table 3:** Diastolic functional class of the rapamycin and placebo groups at baseline, 6 months, and 12 months into the study.

Treatment group	Diastolic functional class*	Baseline	6 months	12 months
Rapamycin	Class 1	4 (44.4)	4 (44.4)	6 (66.7)
	Class 2	3 (33.3)	4 (44.4)	0 (0.0)
	Class 3	2 (22.2)	0 (0.0)	3 (33.3)
	Class 4	0 (0.0)	0 (0.0)	0 (0.0)
Placebo	Class 1	0 (0.0)	2 (25.0)	2 (25.0)
	Class 2	4 (50.0)	2 (25.0)	4 (50.0)
	Class 3	4 (50.0)	4 (50.0)	2 (25.0)
	Class 4	0 (0.0)	0 (0.0)	0 (0.0)

Categorical variables reported as number (%). *Diastolic functional classes are defined as normal pattern (Class 1), relaxation delay pattern (Class 2), pseudonormal pattern (Class 3), and restrictive pattern (Class 4) (55).

### 3.4. Clinicopathologic evaluation

There were no clinically significant laboratory abnormalities among enrolled dogs. All enrolled dogs were seronegative for *Trypanosoma cruzi*. A total of nine dogs (*n* = 5 rapamycin) had serum lipemia detected at one or more visits with a total of 17 instances, including four dogs (*n* = 1 rapamycin) with lipemia at baseline. A total of nine dogs (*n* = 5 rapamycin) had a platelet count below the reference interval detected at one or more visits, with a total of 15 instances. There were only three instances (*n* = 2 rapamycin) at which a platelet count <100,000/μL was documented, and in each instance 4+ clumping was noted on the blood smear.

Mean HOMA-IR scores were not significantly different between control and rapamycin treated groups at baseline (2.35 ± 2.1 and 2.14 ± 1.4, respectively; *p* = 0.82), nor at 6 months (2.61 ± 3.1 and 1.99 ± 1.3; *p* = 0.59). Additionally, the mean change in HOMA-IR over 6 months was not significantly different between rapamycin and control groups (+0.26 ± 2.4 and − 0.15 ± 1.5; *p* = 0.67).

Ultrasensitive cardiac troponin I values for each dog at the baseline and 6 month time points are presented in Supplementary Table 1. Seven of eight placebo dogs were within normal reference ranges for aging dogs at baseline and 6 months (0.0–0.12 ng/mL), as were seven of nine rapamycin-treated dogs. One dog in the rapamycin group had missing troponin samples. One placebo and one rapamycin dog had mildly elevated troponin levels at baseline that were unchanged at 6 months. Finally, one rapamycin dog that originally had a troponin value in the normal range for aging dogs (0.088 ng/mL) had a mildly elevated troponin value at 6 months (0.164) with no observable cardiac change or outward morbidity observed. This change was outside of the established reference change interval for the assay (61).

### 3.5. At-home observation

A total of 442 biweekly at-home observation surveys were requested, and 392 were received for an overall response proportion of 88.7% (94.0% in the rapamycin group; 82.6% in the placebo group). There were no clinically significant owner-reported adverse events among enrolled dogs. Among dogs in the rapamycin group, a total of one dog (11.1%) experienced one episode of self-limiting diarrhea, five dogs (55.5%) experienced a total of eight episodes of self-limiting vomiting, and two dogs (22.2%) experienced a total of three episodes of poor appetite throughout the study. One dog (11.1%) in the rapamycin group appeared mildly constipated during most of the treatment period and bowel movements returned to normal after the treatment period. This same dog had a change in hair coat during the treatment period characterized by decreased shedding, leading to a thick undercoat and dull topcoat; within the first month following the treatment period she had a period of increased shedding, and her coat became glossy again. Among dogs in the placebo group, a total of three dogs (37.5%) experienced four episodes of self-limiting diarrhea, two dogs (25%) experienced a total of 11 episodes of self-limiting vomiting (one of these dogs had been known to have bilious vomiting intermittently over her life prior to the trial), and no dogs experienced poor appetite throughout the study. In response to the question, “Has your dog experienced any positive changes in behavior or other things that you would associate with good health within the past week? (please explain)” owners of dogs in the rapamycin group replied with a “yes” response in 26.8% of all surveys [*n* = 59 out of 220 submissions, representing six of the nine dogs (66%) in this group] while owners of dogs in the placebo group replied with a “yes” response in 8.1% of all surveys [*n* = 14 out of 172 submissions, representing five of the eight dogs (62.5%) in this group]. This frequency of positive owner-reported outcomes in the rapamycin treatment group was significantly greater than in the placebo group (*p* = 0.04). When asked to describe the nature of the observed changes, owners of two dogs in the rapamycin group (*n* = 0 placebo) described increased activity levels and playfulness throughout the treatment period that did not persist into the post-treatment period.

## 4. Discussion

The major objectives of this study were to assess the impact of 6 months of treatment with low-dose rapamycin on baseline indices of diastolic and systolic cardiac function and to document occurrence of adverse events in healthy, middle-aged companion dogs. No clinically or statistically significant difference in echocardiographic parameters between the rapamycin and placebo groups was observed in the dogs in this study at the 6-month or 12-month recheck. Additionally, no statistically or clinically significant adverse events were observed in the rapamycin-treated dogs in this study. A single rapamycin treated dog developed a mildly elevated cardiac troponin I level at the 6 month timepoint (0.164 ng/mL), however the significance of this remains unknown. The dog exhibited no outward cardiac change, nor any obvious clinical morbidity. Additionally, elevations in cardiac troponin I can also be seen in association with non-cardiac disease (62).

The echocardiography results differ from previous work by our group, which showed significantly higher FS and E/A ratio in dogs treated with a shorter duration but higher dose of rapamycin compared to a placebo group in a randomized controlled trial (44). Dogs in the previous trial received 0.05 mg/kg three times weekly in one rapamycin treatment group and 0.1 mg/kg three times weekly in another rapamycin treatment group (both compared to a placebo group in that trial), whereas dogs in this trial’s rapamycin treatment group received 0.025 mg/kg three times weekly. Previous studies with mice have highlighted that dosing can greatly influence the effects of rapamycin (20). Because the treatment duration was substantially extended from 10 weeks in the prior trial to 6 months in the current trial, we chose the lower dose of 0.025 mg/kg three times weekly out of caution for potential adverse events. Clinically significant adverse events were not seen in the dogs of this report, even over the longer duration of the study. Additionally, dogs in the previous trial had a mean age of 9.7 years, while the mean age in the present trial was 7.8 years for the treatment group and 8.5 years for the placebo group. The failure to demonstrate differences in echocardiographic parameters between treatment groups in the study reported here could indicate that the dose in the present study was too low or could relate to the prior trial enrolling an older cohort of dogs with more potential for pre-existing age-related cardiac dysfunction. Additionally, the small number of dogs enrolled decreased the statistical power of the study. The diversity of the breeds enrolled may have been a contributing factor as well.

The sensitivity of different echocardiographic measurements to detect between-group differences in cardiac structure and function should also be considered when interpreting the lack of significant differences in echocardiographic parameters in this study. For instance, while most age-related cardiac declines relate to impaired diastolic function, a concurrent decline in systolic function is not traditionally expected (3, 5). This is evidenced by preserved echocardiographic measurements such as EF in aging human populations (4, 63). More subtle assessments of systolic function such as strain, strain rate, twist or torsion of the left ventricle, however, have demonstrated statistically significant age-related declines in humans (4, 63, 64). Through this lens, the lack of significant differences in EF and FS between treatment groups in this study may be expected. Further study using more sensitive assessments of systolic function, and additional markers of diastolic function beyond E/A ratio and IVRT, may be indicated.

A total of five dogs in the rapamycin group (and four in the placebo group) were reported to have mild and transient thrombocytopenia at least once during the study. A total of five dogs in the rapamycin group (and four in the placebo group) were reported to have visible lipemia at least once during the study. Increased serum cholesterol concentrations were not associated with lipemia in these dogs and serum triglycerides were not measured as part of the routine chemistry profile in this study. As thrombocytopenia (65) and hyperlipidemia (40, 66) are known adverse events in humans treated with rapamycin and other mTOR inhibitors, continued monitoring of these parameters in future studies of rapamycin in dogs is indicated.

Two separate mTOR complexes, mTORC1 and mTORC2, are essential in nutrient-sensing pathways responsible for cell growth and proliferation, and their inhibition can lead to changes in metabolism including glucose regulation (67). Although rapamycin is a specific inhibitor of mTORC1, chronic treatment with rapamycin can lead to indirect inhibition of mTORC2, which has been proposed to cause insulin resistance and impaired response to a glucose tolerance test (68). Surprisingly, however, long-lived mice treated with rapamycin throughout life do not show evidence of impaired glucose homeostasis such as elevated fasting glucose levels or HbA1c, leading to the hypothesis that, on balance, the metabolic changes associated with rapamycin are neutral or beneficial (69, 70). It is difficult to accurately predict what effect rapamycin administration may have on a dog’s glucose regulation, as it likely depends on a combination of factors such as age, duration of treatment, and genetics. For this reason, it was important to investigate potential changes in glucose tolerance in the dogs in this study, and no overt impact on HOMA-IR scores was detected. Continued attention to glucose regulation is warranted in future studies of rapamycin in dogs.

One of the most notable differences in the collected data was that significantly more of the biweekly at-home surveys from rapamycin-treated dogs indicated a “yes” response to the question, “Has your dog experienced any positive changes in behavior or other things that you would associate with good health within the past week? (please explain),” versus a “yes” response to this question in the biweekly at-home surveys from placebo-treated dogs. Despite the increased frequency with which owners reported positive changes in dogs in the rapamycin group, it is worth noting that the number of dogs whose owners reported “positive changes” was similar between groups (six out of nine rapamycin-treated dogs, and five out of eight placebo-treated dogs). Owners who indicated “yes” were given a free-text box in which to describe the observed positive changes. Because of the subjectivity associated with free-text responses, it is difficult to specifically characterize these positive changes. However, given the statistically significant difference in frequency with which this response was given between groups, it will be of interest to investigate the specific positive changes in behavior or health that owners are perceiving as associated with rapamycin treatment in future studies. Interestingly, owners had reported similar subjective benefits to their dogs in our prior masked, placebo-controlled, randomized clinical trial of rapamycin, including increases in owner-reported affection and activity (44).

One of the key challenges of this clinical trial was the process of recruiting dogs that simultaneously met the size and age inclusion criteria without displaying any of the health-related and other exclusion criteria. This was reflected in our high proportion of excluded dogs, with only 17/79 dogs whose owners received the initial online eligibility questionnaire, and only 17/56 dogs whose owners submitted medical records, proceeding to randomization. Because the study population was intended to include “normal” older dogs, it becomes important to define what normal aging is. Many have attempted to clearly define the characteristics of both normal and abnormal aging in dogs (71, 72). These distinctions become increasingly important for this study and others like it that seek to recruit a “normal” aging dog population and to evaluate the ability of novel interventional therapies to mitigate age-related physical and/or pathophysiologic changes. It is possible that our inclusion and exclusion criteria led to the enrollment of an atypically healthy cohort of dogs in this size and age range, and that our results may not be generalizable to a more typical population of dogs.

Another limitation of this study is that left atrial volume was not consistently obtained. In humans, volumetric measurements of left atrial size are considered standard due to their ability to assess for enlargement in multiple planes, rather than the single-dimension LA:Ao ratio that is commonly used in veterinary medicine (1, 2, 73). Because left atrial volume was not analyzed for the dogs in this study, it is possible that subtle changes in left atrial size could have been missed. Additionally, echocardiographic strain measurements were also not consistently obtained in this study. Had it been possible to analyze strain data, more subtle alterations in systolic function may have been identified. Further work is needed to better define the spectrum of expected age-related changes in cardiac structure and function in older dogs, as well as the ideal echocardiographic measurements to detect and monitor those changes over time.

The initial study design sought to enroll 50 dogs (25 placebo, 25 rapamycin) based on power calculations. Several factors caused us to cease enrollment early, limiting the power to detect significant changes in the primary endpoint. These factors included difficulty identifying eligible dogs near the clinical site and the launch of the larger, multi-center clinical trial described below.

In order to further evaluate the effects of rapamycin in the aging dog, our group is conducting a larger masked, placebo-controlled, randomized clinical trial (the Test of Rapamycin in Aging Dogs) in which the rapamycin treatment group will receive a higher cumulative dose (0.15 mg/kg) administered once weekly. There is evidence that intermittent administration of rapamycin yields beneficial effects of lifespan extension in mice (74) and reduces the incidence of adverse events in people (40).

In conclusion, the present study did not show evidence that rapamycin, at the dose and duration given, resulted in any clinically or statistically significant improvement in cardiac function over 12 months as measured echocardiographically. At the same time, there were no clinically significant adverse events associated with rapamycin detected in medical histories, physical examinations or clinical pathology parameters. Some owners reported subjective “positive changes” associated with rapamycin therapy. If rapamycin truly has benefit for cardiac function, the relatively small sample size or lower dose compared with our previous study could be responsible for the lack of statistically significant findings. Further assessment evaluating a higher dosing regimen with a larger study population may be more effective at identifying a beneficial effect of rapamycin on cardiac health in dogs.

## Data availability statement

The raw data supporting the conclusions of this article will be made available by the authors, without undue reservation.

## Ethics statement

The animal study was reviewed and approved by the Texas A&M University School of Veterinary Medicine and Biomedical Sciences Institutional Animal Care and Use Committee (IACUC) and Clinical Research Review Committee protocol number 2017–0125. Written informed consent was obtained from the owners for the participation of their animals in this study.

## Author contributions

BB, KC, and SW wrote the initial draft of the manuscript. KC, DP, MK, SW, SG, AS, LC, and JE developed the experimental design and were involved in data collection. SS performed analysis of the study data. All authors had the opportunity to participate in editing both the form and content of the manuscript and approved the final version.

## Funding

This study was funded by the William H. Donner Foundation. Additional support for some authors was provided by the Dog Aging Project (NIA U19 AG057377; BB, DP, MK, JE, LC, and KC).

## Conflict of interest

The authors declare that the research was conducted in the absence of any commercial or financial relationships that could be construed as a potential conflict of interest.

## Publisher’s note

All claims expressed in this article are solely those of the authors and do not necessarily represent those of their affiliated organizations, or those of the publisher, the editors and the reviewers. Any product that may be evaluated in this article, or claim that may be made by its manufacturer, is not guaranteed or endorsed by the publisher.
